# Effects of fee-for-service, diagnosis-related-group, and mixed payment systems on physicians’ medical service behavior: experimental evidence

**DOI:** 10.1186/s12913-022-08218-5

**Published:** 2022-07-05

**Authors:** Xing Li, Yue Zhang, Xinyuan Zhang, Xinyan Li, Xing Lin, Youli Han

**Affiliations:** School of Public Health, Capital Medical University, No.10 Xitoutiao, Youanmenwai Street, Fengtai District, Beijing, 100069 China

**Keywords:** Mixed payment, Physicians’ behavior, Experimental economics

## Abstract

**Background:**

Healthcare reforms in many countries have shown a movement from pure payment systems to mixed payment systems. However, there remains an insufficient understanding of how to design better mixed payment systems and how such systems, especially Diagnosis-Related-Group (DRG)-based systems, benefit patients. We therefore designed a controlled laboratory experiment to investigate the effects of fee-for-service (FFS), DRG, and mixed payment systems on physicians’ service provision.

**Methods:**

A total of 210 medical students were recruited from Capital Medical University as subjects. They, in the role of physicians, were randomly divided into seven groups and chose the quantity of medical services for different patient types under pure FFS, pure DRG, or mixed payment schemes that included two FFS-based mixed payment schemes and three DRG-based mixed payment schemes. There were five rounds of each group of experiments, and each subject made 18 decisions per round. The quantity of medical services provided by subjects were collected. And relevant statistics were computed and analyzed by nonparametric tests and random effects model.

**Results:**

The results showed that the physicians’ overprovision (underprovision) of services under FFS (DRG) schemes decreased under mixed payment schemes, resulting in higher benefit to patients under mixed payment schemes. Patients’ health conditions also affected physicians’ behavior but in different directions. Higher disease severity was associated with higher deviation of physicians’ quantity choices from the optimal quantity under DRG and DRG-based mixed payment schemes, while the opposite was found for FFS and FFS-based mixed payment schemes.

**Conclusions:**

Mixed payment systems are a better way to balance physicians’ profit and patients’ benefit. The design of mixed payment systems should be adjusted according to the patient’s health conditions. When patients are in lower disease severity and resource consumption is relatively small, prospective payments or mixed systems based on prospective payments are more suitable. While for patients in higher disease severity, retrospective payments or mixed systems based predominantly on retrospective payments are better.

**Supplementary Information:**

The online version contains supplementary material available at 10.1186/s12913-022-08218-5.

## Introduction

A balance between controlling health expenditures and maintaining medical quality is an important issue in healthcare reform. Reforming payment systems is considered to be an effective way to achieve such a balance [1]. Thus, the choice of payment method is a crucial decision in healthcare reform. Payment methods can be retrospective or prospective, depending on whether the payment rate for a unit of service is set retrospectively or prospectively. Retrospective payment methods (e.g., fee for service; FFS) are not conducive to controlling medical costs because such methods incentivize physicians to overprovide care to increase their income; meanwhile, prospective payment methods (e.g., capitation; CAP) can help control medical costs but might lead to the underprovision of care (e.g., [2, 3]) . To overcome the weaknesses of the pure payment methods, mixed payment methods that combine prospective and retrospective methods have become a reform pathway. Ellis and McGuire [4] developed a model in which physicians chose the quantity of service provided to patients, and their provision behavior affected their income as well as the benefit for patients. They identified an overprovision and underprovision of service under retrospective and prospective payment, respectively, and proposed a transformation from pure payment methods to mixed payment methods to optimize medical service. Other theoretical studies have also found that mixed payment methods can overcome the weaknesses of pure payment methods, thus providing theoretical support for using mixed payment methods [5–8].

Healthcare reforms in many countries (e.g., Canada, Japan, South Korea) have shown a trend toward replacing pure payment methods with mixed payment methods that combines FFS and fixed payment (e.g., CAP, per bed, per case, or per diem) [9–11]. The Diagnosis-Related-Group (DRG) is a kind of patient classification system that divides patients into economically and clinically similar groups [12]. When a hospital treats a patient in a certain DRG category, the fee paid to the provider is fixed, regardless of actual medical expenditure [13]. In terms of its predetermined fixed rate and strong incentives for cost containment, DRG is usually regarded as a prospective payment method. Many countries, especially low- and middle-income countries, increasingly use DRG-based payment systems to remunerate healthcare providers [14]. By the end of 2021, China’s basic medical insurance had covered 1.36 billion people, thus making its healthcare system one of the largest worldwide. Under the background of universal medical insurance, the double challenges of rapid rise of health expenditure and ensuring the quality of medical care make the Chinese government start the reform of payment methods. The Chinese government has been exploring and implementing multiple payment methods since 2009, and DRG payment has become an important alternative to conventional FFS payment.

As physicians are direct providers of medical services, their behavioral responses to payment methods can determine the effect of payment system reform. With this in mind, many researchers have investigated the relationship between payment methods (mainly FFS, CAP, and pay-for-performance; P4P) and physicians’ medical service behavior. Although such studies have supported the abovementioned theoretical findings (e.g. [15–17]) , some have found no strong relationship between payment methods and physicians’ medical service provision (e.g., [18–20]). Furthermore, the empirical evidence is mixed as to whether mixed payment methods are better in containing costs and maintaining healthcare quality than pure payment methods. Zhang and Sweetman [9] found that the Canadian blended capitation (CAP-FFS) payment incentives led general practitioners to provide more FFS services. Fu et al. (2021) [21] found that the reform from pure FFS to mixed payment system did not lead to reduction in cost, even indicated a decline in the probability of symptoms being cured at discharge. And, there are not many studies focusing on the effects of reform from pure FFS to DRG-based payment methods on physicians’ behavior.

It is challenging to study the effects of payment methods on physicians’ behavior empirically because of the difficulty in making causal inferences about the direction and intensity of the incentive effects of payment methods. Differences in healthcare systems and heterogeneity of physicians’ intrinsic motivations further complicate this issue [22]. Because of these problems, some studies have recently used laboratory experiments to investigate how physicians respond to payment methods. Hennig-Schmidt et al. [23] designed a controlled laboratory experiment to investigate the effects of FFS and CAP on physicians’ supply of medical services. In the experiment, medical students, as subjects, chose the quantity of medical service given to patients, and their choices determined their own profits as well as the level of benefit for patients. The results of this study provided support for the theoretical predictions of Ellis and McGuire [4]. Most subsequent experimental studies of the effects of payment incentives on physicians’ behavior have followed the design used by Hennig-Schmidt et al. [23]. Extension studies have investigated the effects of mixed payment methods combining FFS and CAP [24, 25] and P4P [26–28] schemes. Others have used “real effort” experiments to investigate how physicians respond to payment methods, mainly including FFS, CAP, P4P, and salary [29–31]. Few studies, however, have studied the effects of DRG and DRG-based mixed payment methods on physicians’ medical service provision. Although these issues were partly covered by Xi et al. [32] and Zhang et al. [33], they only considered the incentive effects of pure DRG.

Based upon the methodologies reported by Brosig-Koch et al. [24, 25], we modified the experimental design and extended it in two aspects. First, we adjusted and refined the experimental parameters according to the characteristics of FFS and DRG to ensure that the experimental conditions were closer to reality. Second, we included five different mixed payment schemes that combined FFS and DRG in different ratios, in particularly, we designed three DRG-based mixed payment schemes, which was not available in the previous experiments. Our experiment aimed to answer the following questions: (1) Do physicians’ behavior and related patients’ benefit improve from pure FFS or pure DRG schemes to mixed payment schemes? (2) Are physicians’ provision behavior and related patients’ benefit different among different mixed payment schemes?

## Methods and experimental setting

### Experimental protocol

The computerized experiment was programmed using Z-tree [34] and conducted in October 2020. We used G*power 3.1.9.7 [35] to calculate the sample size with a power of 80%, a 5% significance level and an effect size of 0.5 [36], and the results showed that it needed at least 23 physicians per group. Considering the experimental operability and the sample size of related economic experiments on payment systems (e.g. [23,24,25]), we determined the sample size was 30 subjects per group. We recruited 210 medical students of Capital Medical University as subjects and they were randomly divided into seven groups (see Table 1). All the subjects had entered hospitals to do clinical rotations and over 40% subjects had started standardized resident training in hospitals. These subjects had medical knowledge and clinical practice, which could make them understand our experimental content better. There was no difference in the distribution of age, gender, and education among the different groups (*p* > 0.05) (see Table 2).

**Table 1:** Experimental groups

Group	Experimental Condition	Part 1	Part 2	Subjects
I	A-D2	DRG	Mix-more-DRG(2)	30
II	A-D4	DRG	Mix-more-DRG(4)	30
III	A-D6	DRG	Mix-more-DRG(6)	30
IV	A-F8	FFS	Mix-more-FFS(8)	30
V	A-F6	FFS	Mix-more-FFS(6)	30
VI	P-NA-D2	DRG^pre^	NA-Mix-more-DRG(2)	30
VII	P-NA-F8	FFS^pre^	NA-Mix-more-FFS(8)	30

This table shows all experimental conditions and the number of subjects in our experiment. The Mix-more-DRG (FFS) means higher DRG (FFS) weight in mixed payment schemes. A-D2, A-D4, A-D6 are the pure DRG, adjusted Mix-more-DRG(2), Mix-more-DRG(4), Mix-more-DRG(6). A-F8 and A-F6 are the pure FFS, adjusted Mix-more-FFS(8) and Mix-more-FFS(6). P-N-D2: the presentation of pure DRG (DRG^pre^) and non-adjusted Mix-more-DRG(2) (NA-mix-more-DRG(2)). P-N-F8: the presentation of pure FFS (FFS^pre^) and non-adjusted Mix-more-FFS(8) (NA-mix-more-FFS(8)). The group VI and group VII were designed to test whether the difference in presentation of the payment schemes (pure payment schemes or mixed payment schemes) affected physicians’ behavior. The results on the “presentation effects” in group VI and group VII were reported in the “Additional file 4”

*DRG* Diagnosis-Related-Group, *FFS* Fee-for-Service

**Table 2:** Distribution of age, gender, and education among different groups

Group	Age	Gender		Education	
	Mean (SD)	Male	Female	Undergraduates	Graduates
I	22.00 (2.05)	7	23	17	13
II	21.77 (2.11)	14	16	17	13
III	22.33 (2.14)	8	22	17	13
IV	22.30 (2.64)	10	20	17	13
V	22.07 (2.30)	5	25	17	13
VI	22.57 (2.58)	8	22	17	13
VII	21.93 (2.02)	7	23	17	13

This table shows the distribution of age, gender, and education among different groups. The column 2 shows the average age of subjects in each group. The columns 3 to 6 show the number of subjects in different genders and educational backgrounds in each group

The experiments each subject participated in consisted of two parts. Each subject i, in the role of physician, was required to choose a quantity of medical service, q ∈ {0, 1, …, 10}, for nine different types of patients under given experimental conditions (Part 1: pure FFS or pure DRG; Part 2: mixed payment schemes) in each part. The types of patients differed in the type of illness, k ∈ [A, B, C], and the severity of illness, j ∈ [moderate (l), intermediate (m), severe (h)]. It was assumed that all patients had medical insurance and accepted any medical service provided by physicians. Based on their service quantity choices, physicians received a certain payment R and also incurred costs C_(q)_ = 0.1·q^2^ [5] for treating the patients. The quantities provided by physicians determined their own profit π_kj_^i^ (R − C_(q)_) as well as patient benefit B_kj_(q). The quantity corresponding to the maximum patient benefit was the optimal quantity, q*, which depended on the severity of illness. When severity was l, m, h, q* was 3, 5, 7, respectively (e.g. [23–27]). Taking q* as a benchmark, we could judge whether the quantity of medical service provided by the subjects amount to overprovision or underprovision. Decisions made by the subjects in any part of the experiment did not affect other parts of the experiment. In order to ensure the robustness of results, we took a five-round experimental design based on our pre-test experiment. The subjects were exposed to pure payment schemes in the first part of the experiment followed by mixed payment schemes in the second part and it repeated five times.

The procedure was as follows. First, subjects were randomly allocated to different computers. Then, they were given enough time to read the experimental instructions and signed informed consent. The subjects were informed that the patient benefit in the experiment would be donated to help real patients. The subjects were not allowed to communicate with each other. If they had questions, they could raise their hands, and an investigator would answer the questions in private. Before the experiment, the subjects had to answer some control questions and complete a pilot experiment. The subjects could only participate in the experiment after they had answered the control questions and completed the pilot experiment correctly (see Additional file 1). In each part of the experiment, subjects decided the q for nine types of patients according to information presented on the computer screen. Taken into account the operability of our experiments based on previous researches (e.g. [23, 37]), the order of the types of patients was predetermined—specifically, A_l_, B_l_, C_l_, A_m_, B_m_, C_m_, A_h_, B_h_, C_h_ and it kept consistent for all the subjects in all experimental conditions. After the subjects completed the decision-making tasks, they were asked to fill out a questionnaire. The questionnaire covered areas such as the reasons for participating in the experiment, the determinants of decision-making, and feelings about the whole experiment (see Additional file 2).

The experiments lasted about an hour and a half. And 18,900 (9∙5∙2∙210) units of experimental records were collected in total. Talers were used as the experimental currency; 1 Taler = 0.1 CNY. Subjects received a sum of π(q) for five rounds plus a basic reward of 30 CNY for participating; each subject earned 108 CNY on average. The sum of B(q) for one of the five rounds, which we chose randomly, was donated to the Red Cross Society of China. To ensure the authenticity of the donation, we randomly selected two of the subjects as monitors. After the experiment, the monitors verified that 3420 CNY was transferred to the Red Cross Society of China through the financial department of the Capital Medical University. And each monitor could earn an additional 50 CNY.

### Experimental conditions

#### Pure payment schemes

Under FFS, physicians’ total compensation is R = pq, which is based on the fee, p, physicians receive for each service they provide. Accordingly, physicians’ profit is π_kj_^i^ = R − C_(q)_ = pq − 0.1·q^2^. Considering the maximum patient benefit and the consistency of physicians’ maximum profit under pure FFS and FFS-based mixed payment schemes, we set the p of illnesses A, B, and C as 1.91, 2, and 2.1, respectively. q^, the chosen quantity to maximize physicians’ profit, is 10 under FFS. Thus, the maximum physicians’ profits for illnesses A, B, and C are 9.1, 10, and 11, respectively.

Given the complexity of DRG classification, we simplified it based on its characteristics and designed a specific DRG to facilitate the experiments. A specific DRG is similar to the per capita of multiple disease groups. This means that physicians can get different fixed compensation fees for different disease groups and the classification of disease groups depends on the types of diseases and their severity. Hence, physicians receive a lump-sum payment (R = LS) per patient type based on k and j under DRG. Considering the relationship between DRG and FFS, we take the R of q* under FFS as LS. Taking illness A as an example, the optimal quantities are q_Al_ = 3, q_Am_ = 5, and q_Ah_ = 7, and the corresponding physicians’ payments are 1.91 × 3 = 5.73(A_l_), 1.91 × 5 = 9.55(A_m_), and 1.91 × 7 = 13.37(A_h_), respectively. Similarly, the LS of B_l_, B_m_, B_h_, C_l_, C_m_, and C_h_ are 6, 10, 14, 6.3, 10.5, and 14.7, respectively. The maximum physicians’ profit is equal to LS because q^ is 0 under DRG.

#### Mixed payment schemes

Mixed payment schemes consisted of DRG and FFS in different proportions. The DRG (FFS)-based mixed payment schemes were labeled as Mix-more-DRG (FFS) schemes. To ensure the comparability of pure and mixed payment schemes, the maximum profit of physicians π(q^) under the mixed payment schemes was the same as that under the pure payment schemes. Assuming the weight of DRG is μ, physicians’ profit π_kj_^i^(q) = μLS + (1 − μ) pq − 0.1·q^2^. This can be transformed into π_kj_^i^(q) = − 0.1·q^2^ + (1 − μ) pq + μLS. Based on the characteristics of unitary quadratic equations, when q = 5p (1 − μ), the physician gets the maximum profit.

Considering that physicians’ service provision is lower under DRG, we assume the q^ under Mix-more-DRG schemes are 2, 4, and 6, which is less than the q* under different disease severities. We therefore designed three schemes with more weight on DRG, Mix-more-DRG(2), Mix-more-DRG(4), and Mix-more-DRG(6). For example, in the Mix-more-DRG(2) scheme, π(q^) = 9.55 = μ·9.55 + (1 − μ)·19.55–0.1·q^2^, substituting q = 2 to get μ = 0.96. To reflect the LS differences for different severities under Mix-more-DRG schemes, the μ of A_l,_ A_m_, and A_h_ were set to 0.97, 0.96, and 0.95, respectively. Moreover, to equalize the π(q^) in the Mix-more-DRG schemes to that of the pure DRG, we adjusted the p and LS. Taking A_l_ as an example, *p* = 2 / 5 × (1–0.97) = 13.33, π(q^) = 0.97·LS + 0.03 × 13.33 × 2–0.4 = 5.73, and the value of LS is 5.49. Similarly, the weights of DRG under Mix-more-DRG(4) and Mix-more-DRG(6) in severity m are 0.84 and 0.64, respectively. The p and LS under Mix-more-DRG schemes can be calculated with reference to the examples.

In the Mix-more-FFS schemes, we put more weight on FFS and set two schemes: Mix-more-FFS(8) and Mix-more-FFS(6). The weights of FFS under Mix-more-FFS(8) and Mix-more-FFS(6) schemes with severity m are 0.80 and 0.60, and the q^ are 8 and 6, respectively; this is similar to the design in Brosig-Koch et al. [24]. The weights of FFS under severity l (h) are 0.79 (0.81) and 0.59 (0.61) under Mix-more-FFS(8) and Mix-more-FFS(6), respectively. To ensure that π(q^) was the same under Mix-more-FFS schemes and pure FFS, we adjusted LS. Taking A_l_ under the Mix-more-FFS(6) scheme as an example, π(q^) = 0.41·LS + 0.59 × 1.91 × 6–3.6 = 9.1, and the value of LS is 14.48. Similarly, LS under Mix-more-FFS schemes can be calculated with reference to the above example.

From the pure DRG (FFS) to the Mix-more-DRG (FFS) schemes, q^ changes from 0 (10) to 2, 4, and 6 (8 and 6). q^ in mixed payment schemes is closer to q* than that in pure payment schemes. This reduces the trade-off between physicians’ profit and patients’ benefit; thus, the physicians’ behavior under mixed payment schemes is more likely to improve the benefit for patients.

To ensure that π(q^) under the pure and mixed payment schemes was the same, we adjusted the p and LS of mixed payment schemes. If we didn’t adjust the p and LS (referred to as “non-adjusted mixed payment schemes”), π(q^) would have been reduced. That was, the financial incentive under non-adjusted mixed payment schemes was slightly lesser than that under adjusted mixed payment schemes. Some previous studies have found that payment levels affected the provision of medical services, and the reduction of payment levels led to a decrease in the intensity of care [38]. A question arises as to whether the decrease in physicians’ maximum profit under non-adjusted mixed payment schemes influences their behavior. Thus, we investigated the effect of payment levels on physicians’ behavior by comparing physicians’ quantity choices under adjusted and non-adjusted mixed payment schemes.

Two non-adjusted mixed payment schemes were designed and labeled as NA-Mix-more-DRG(2) and NA-Mix-more-FFS(8). The weight of DRG (FFS) under the two non-adjusted mixed payment schemes is the same as that under Mix-more-DRG(2) and Mix-more-FFS(8). Taking A_l_ as an example, if p and LS are not adjusted, π(q^) is 5.27 under Mix-more-DRG(2), and π(q^) is 6.87 under Mix-more-FFS(8). In NA-Mix-more-DRG(2), to ensure π(q^) is 5.27 at q = 2, we need to adjust the cost function C_(q)_. According to 0.97 × 5.73 + 0.03 × 1.91·q − C_1(q)_ = 5.27 = − 0.1·(q − 2)^2^ + 5.27, and the solution is C_1(q)_ = 0.1·q^2^–0.3427·q + 0.6881. Similarly, in the NA-Mix-more-FFS(8), the cost function C_(q)_ should also be adjusted to make sure π(q^) is 6.87 at q = 8. According to 0.21 × 5.73 + 0.79 × 1.91·q − C_2(q)_ = 6.87 = − 0.1·(q − 8)^2^ + 6.87, and the solution is C_2(q)_ = 0.1·q^2^–0.0911·q + 0.733. The other adjusted cost function could be calculated with reference to the example A_l_.

#### Patient benefit

The patient benefit was determined as B_kj_(q) = B_kj_(q*) − θ|q − q*| [27]. q*, the optimal quantity; θ refers to the marginal patient benefit; θ_A_ = θ_B_ = 1, θ_C_ = 2. The maximum patient benefit, B_kj_(q*), under different illnesses was different; B_Aj_(q*) = 7, B_Bj_(q*) = 10, B_Cj_(q*) = 14 [24, 25]. The specific parameters of patient benefit see Additional file 3.

#### Statistical analyses

We analyzed the differences in physicians’ behavior and patient benefit through nonparametric analysis. Specifically, the matched-pairs Wilcoxon signed-rank (WSR) test was used for comparison at the aggregate level or in different disease severities between two parts in each group; the Mann–Whitney U (MWU) test and the Kruskal–Wallis H (KWH) test were used for comparison between groups; and the Friedman test was used for comparison among different disease severities under the same payment scheme conditions. The Bonferroni correction was used in the post-hoc tests of the KWH test and Friedman test. All the above tests were two-sided, and the significance level was set at 0.05.

The random effects model was used to test for the robustness of effects of mixed payment schemes and other control conditions on physicians’ behavior. The model is Y_it Lkj_ = β_0_ + β_1_Payment (DRG, FFS) _it_ + β_2_k_it_ + β_3_j_it_ + λZ_i_ + u_i_ + ε_it_, where L_kj_ is the loss of patient benefit, which is calculated as follows: L_kj_ = ((B(q*) − actual patient benefit) / B(q*)). Payment (DRG, FFS) is a set of dummy variables for DRG(FFS)-based payment schemes, k and j are type of illness and severity of illness, Z_i_ is a vector of individual characteristics, u_i_ is individual-specific effect that does not vary over time and ε_it_ is an error term. Robust standard errors are clustered at the individual subject level.

## Results

### Provision behavior under pure payment schemes

Aggregate data of physicians’ quantity choices under pure payment schemes is shown in Table 3. In groups I, II and III, we used the same DRG payment scheme in Part 1 of every group. Although physicians’ quantity choices under pure DRG differed among the above three groups at the aggregate level (*p* < 0.001, KWH test; further pairwise comparison: I vs II, I vs III, II vs III, adjusted *p* < 0.05), there were no significant differences in the physicians’ quantity choices among the three groups at the patient level (patient A_l_, B_l_, C_l_, A_m_, B_m_, C_m_, A_h_, B_h_, C_h_: *p* > 0.05 except for A_l_, A_h_, B_h_, C_h_, KWH test; further pairwise comparision showed that the differences were not significant). As such, we aggregated the data in these pure DRG conditions in our analysis of pure payment schemes. The average quantity of medical service was 3.71 under DRG in general. For groups IV and V, we used the same FFS payment scheme in Part 1 in each group. Since there were no significant differences in physicians’ quantity choices under FFS between groups IV and V at either the aggregate level (*p* = 0.472, MWU test) or patient level (patient A_l_, B_l_, C_l_, A_m_, B_m_, C_m_, A_h_, B_h,_ C_h_: *p* > 0.05 except for A_m_, MWU test), we also aggregated the data in these pure FFS conditions in our analysis of pure payment schemes. The average quantity of medical service was 5.82 under FFS in general. The comparison of aggregate data in physicians’ quantity choices between pure DRG and pure FFS indicated that the average quantity of medical service under pure FFS was significantly higher than that under DRG (*p* < 0.001, MWU test).

**Table 3:** Descriptive statistics for quantity choices under pure payment schemes

Group	Experimental Condition	Part 1 (Pure Payment Schemes)	
		Mean	SD
I	A-D2	3.73	1.97
II	A-D4	3.49	1.87
III	A-D6	3.92	1.79
IV	A-F8	5.85	1.68
V	A-F6	5.78	1.81

This table shows the average physicians’ quantity choices under pure payment schemes in Part 1 of each group. Experimental conditions A-D2, A-D4, A-D6 refer to pure DRG in groups I, II and III; A-F8 and A-F6 refer to pure FFS in group IV and V

*DRG* Diagnosis-Related-Group, *FFS* Fee-for-Service

**Fig. 1:**
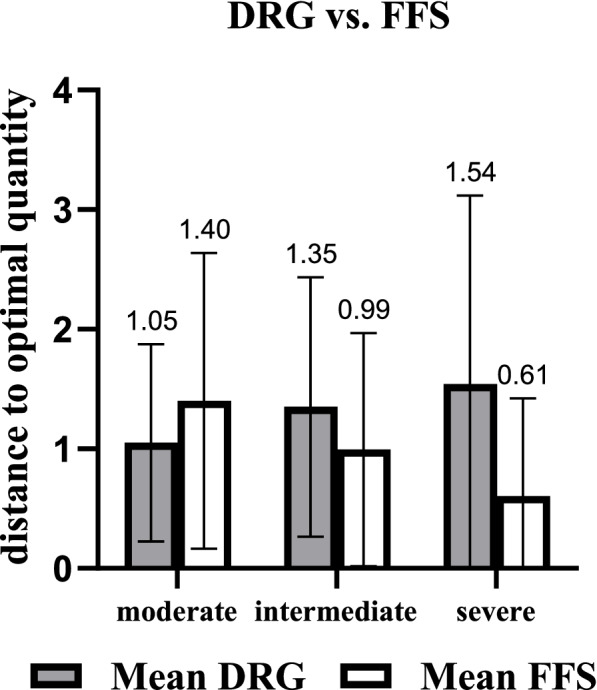
Distance between quantity choices and optimal quantity under pure payment schemes

Comparing the absolute distance between the physicians’ quantity choices and q* in different disease severities under the two pure payment schemes (see Fig. 1) revealed that higher disease severity was associated with greater differences between physicians’ quantity choices and q* under DRG; meanwhile, it was the opposite under FFS (DRG: *p* < 0.001, Friedman test, further pairwise comparisons: moderate vs intermediate, moderate vs severe, both adjusted *p* < 0.001; intermediate vs severe, adjusted *p* = 0.294; FFS: *p* < 0.001, Friedman test, further pairwise comparisons: moderate vs intermediate, moderate vs severe, intermediate vs severe, adjusted *p* < 0.001).

### Comparison of physicians’ behavior under pure and mixed payment schemes

Table 4 shows the aggregate data for physicians’ quantity choices under the mixed payment schemes. Comparing physicians’ quantity choices under pure DRG or pure FFS (in Part 1) with the respective mixed payment schemes (in Part 2) in groups I, II, III, IV and V, there were significant differences between the pure payment schemes and mixed payment schemes. Specifically, there was a higher and lower provision of service under Mix-more-DRG and Mix-more-FFS schemes than under pure DRG and pure FFS, respectively (groups I, II, III, IV and V, *p* < 0.001, WSR test).

**Table 4:** Descriptive statistics for quantity choices under mixed payment schemes

Group	Experimental Condition	Part 2 (Mixed Payment Schemes)	
		Mean	SD
I	A-D2	4.13	1.70
II	A-D4	4.63	1.33
III	A-D6	5.24	1.36
IV	A-F8	5.68	1.61
V	A-F6	5.20	1.54

This table shows the average physicians’ quantity choices under mixed payment schemes in Part 2 of each group. Experimental conditions A-D2, A-D4, A-D6 refer to adjusted Mix-more-DRG(2), Mix-more-DRG(4), Mix-more-DRG(6); A-F8 and A-F6 refer to adjusted Mix-more-FFS(8) and Mix-more-FFS(6)

*DRG* Diagnosis-Related-Group, *FFS* Fee-for-Service

Table 5 shows the average deviation between physicians’ quantity choices and the optimal quantity q* under different payment schemes in groups I, II, III, IV and V. In groups I, II, III, the quantity of medical service under the Mix-more-DRG schemes was closer to q* than that under pure DRG (comparison at aggregate level of each group, *p* < 0.001; group I, group II and group III: moderate, intermediate, severe, p < 0.001; WSR test). In groups IV and V, the quantity of medical service under the Mix-more-FFS scheme decreased compared with that under pure FFS. So, the deviation between the quantity of medical service and q* under Mix-more-FFS schemes was lower (comparison at aggregate level of each group, *p* < 0.001; group IV: moderate, *p* = 0.037 < 0.05; intermediate, severe, both *p* < 0.001; group V: moderate, intermediate, severe, *p* < 0.001; WSR test).

**Table 5:** Deviation between quantity choice and the optimal quantity

Group	Experimental Condition	q − q*	Pure Payment Scheme		Mixed Payment Scheme	
			Mean	SD	Mean	SD
I	A-D2	aggregate level	−1.27	1.33	−0.87	1.04
		moderate	−1.01	0.90	−0.49	0.64
		intermediate	−1.36	1.20	−0.97	0.91
		severe	−1.44	1.72	−1.14	1.35
II	A-D4	aggregate level	−1.51	1.39	−0.37	0.85
		moderate	−1.10	0.86	0.24	0.61
		intermediate	−1.48	1.18	−0.40	0.52
		severe	−1.95	1.83	−0.96	0.89
III	A-D6	aggregate level	−1.08	0.95	0.24	0.76
		moderate	−0.90	0.85	0.82	0.87
		intermediate	−1.16	0.89	0.11	0.46
		severe	−1.17	1.06	−0.22	0.42
IV	A-F8	aggregate level	0.85	1.12	0.68	1.12
		moderate	1.25	1.39	1.16	1.38
		intermediate	0.87	0.90	0.70	0.96
		severe	0.43	0.84	0.17	0.65
V	A-F6	aggregate level	0.78	1.31	0.20	1.10
		moderate	1.19	1.44	0.76	1.28
		intermediate	0.80	1.29	0.21	0.86
		severe	0.35	1.03	−0.37	0.77

This table shows the average deviation between physicians’ quantity choices and the optimal quantity q* under different payment schemes in groups I, II, III, IV and V. A-D2, A-D4, A-D6 are the pure DRG, adjusted Mix-more-DRG(2), Mix-more-DRG(4), Mix-more-DRG(6). A-F8 and A-F6 are the pure FFS, adjusted Mix-more-FFS(8) and Mix-more-FFS(6). “Aggregate level” refers to aggregate data for nine types of patients

*DRG* Diagnosis-Related-Group, *FFS* Fee-for-Service

**Fig. 2:**
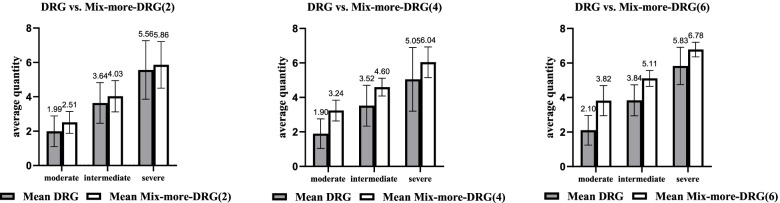
Average quantity choices in DRG and Mix-more-DRG payment schemes

**Fig. 3:**
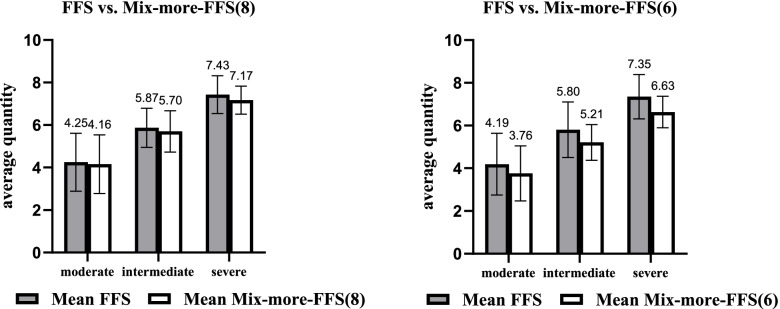
Average quantity choices in FFS and Mix-more-FFS payment schemes

The comparison of physicians’ quantity choices in Part 2 for groups I, II and III showed that higher quantity of medical service was associated with less weight given to the DRG component under Mix-more-DRG schemes (*p* < 0.001, KWH test; further pairwise comparison: I vs II, I vs III, II vs III, adjusted *p* < 0.001). This means that introducing FFS in Mix-more-DRG schemes could improve the underprovision observed under pure DRG (see Fig. 2). The differences in the physicians’ quantity choices in Part 2 for groups IV and V showed that the quantity of medical service decreased with decreasing the FFS component in Mix-more-FFS schemes (*p* < 0.001, MWU test). That is, introducing DRG in Mix-more-FFS schemes could reduce the overprovision caused by pure FFS (see Fig. 3).

### Analysis of non-adjusted mixed payment schemes: payment level effects

The experimental conditions of Part 2 in groups I and VI were adjusted and non-adjusted Mix-more-DRG(2) payment schemes. The experimental conditions of Part 2 in groups IV and VII were adjusted and non-adjusted Mix-more-FFS(8) payment schemes. The reduction of physicians’ maximum profit did not affect physicians’ behavior significantly under adjusted and non-adjusted Mix-more-DRG(2) payment schemes (*p* = 0.014 < 0.05 at the aggregate level; patient A_l_, B_l_, C_l_, A_m_, B_m_, C_m_, A_h_, B_h_, C_h_: *p* > 0.05 except for B_m_; MWU test). The difference in the quantity of medical service under adjusted and non-adjusted Mix-more-FFS(8) payment schemes was statistically significant (*p* < 0.001 at the aggregate level; just patient A_l_, B_l_, A_h_, B_h_, C_h_: *p* < 0.05; MWU test). 

### Analysis of patient benefit under pure and mixed payment schemes

First, we compared patient benefit under the pure and mixed payment schemes for each group. Patients’ benefit under pure payment schemes were lower than under mixed payment schemes in groups I, II, III, IV and V (*p* < 0.001, WSR test) (see Table 6). In addition, there was no significant difference in patients’ benefits between the adjusted and non-adjusted payment schemes (adjusted and non-adjusted Mix-more-DRG(2), *p* = 0.075; adjusted and non-adjusted Mix-more-FFS(8), *p* = 0.039 < 0.05; moderate, *p* = 0.008 < 0.05, intermediate, *p* = 0.243, severe, *p* = 0.839; MWU test).

**Table 6:** Descriptive statistics for patient benefit

Group	Experimental Condition	Pure Payment Scheme		Mixed Payment Scheme	
		Mean	SD	Mean	SD
I	A-D2	8.61	3.00	9.16	2.94
II	A-D4	8.34	3.02	9.57	2.88
III	A-D6	8.88	2.77	9.76	2.83
IV	A-F8	9.04	2.79	9.24	2.86
V	A-F6	8.90	2.75	9.43	2.86

This table shows the average patient benefit under pure payment scheme and mixed payment scheme of each group. A-D2, A-D4, A-D6 are the pure DRG, adjusted Mix-more-DRG(2), Mix-more-DRG(4), Mix-more-DRG(6). A-F8 and A-F6 are the pure FFS, adjusted Mix-more-FFS(8) and Mix-more-FFS(6)

Diagnosis-Related-Group, *FFS* Fee-for-Service

Table 7 showed that the L_kj_ under different mixed payment schemes in groups I, II, III, IV and V. The random effects model (REM) was used to analysis the loss of patient benefit under different payment schemes. Panel A (columns 1 to 3) and Panel B (columns 4 to 6) in Table 8 showed regression results for DRG-based schemes and FFS-based schemes, respectively. The dependent variable loss was L_kj_. The results supported that the L_kj_ was reduced under mixed payment schemes compared to pure payment schemes. In Mix-more-DRG schemes, the L_kj_ decreased most in Mix-more-DRG(4) payment scheme. The L_kj_ in Mix-more-FFS schemes decreased most in Mix-more-FFS(6) payment scheme. After k and j were controlled, the L_kj_ in illness B (B(q*) increased) and illness C (both B(q*) and θ increased) decreased in DRG-based payment schemes compared with illness A; the L_kj_ decreased in illness B but increased in illness C in FFS-based payment schemes compared with illness A. And the L_kj_ increased (decreased) in DRG (FFS)-based payment schemes as disease severity increased.

**Table 7:** Loss of patient benefit based on disease severity under mixed payment schemes

Group	Mixed Payment Schemes	Moderate		Intermediate		Severe	
		Mean	SD	Mean	SD	Mean	SD
I	A-D2	0.07	0.08	0.13	0.11	0.15	0.17
II	A-D4	0.05	0.07	0.05	0.07	0.12	0.11
III	A-D6	0.11	0.11	0.03	0.05	0.03	0.05
IV	A-F8	0.17	0.16	0.10	0.11	0.04	0.08
V	A-F6	0.13	0.14	0.07	0.09	0.06	0.09

This table shows the average loss of patient benefit under mixed payment schemes in group I, II, III, IV and V. Experimental conditions A-D2, A-D4, A-D6 refer to adjusted Mix-more-DRG(2), Mix-more-DRG(4), Mix-more-DRG(6); A-F8 and A-F6 refer to adjusted Mix-more-FFS(8) and Mix-more-FFS(6)

Diagnosis-Related-Group; *FFS* Fee-for-Service

**Table 8:** REM regression of the loss of patient benefit

Independent variable	DRG			FFS		
	(1)	(2)	(3)	(4)	(5)	(6)
**Payment: DRG/FFS (ref)**						
Mix-more-DRG(2)	−0.053***	−0.053***	−0.053***			
	(0.009)	(0.009)	(0.009)			
Mix-more-DRG(4)	−0.118***	− 0.118***	− 0.118***			
	(0.017)	(0.017)	(0.017)			
Mix-more-DRG(6)	−0.088***	− 0.088***	− 0.088***			
	(0.011)	(0.011)	(0.011)			
Mix-more-FFS(8)				−0.019**	−0.019**	−0.019**
				(0.006)	(0.006)	(0.006)
Mix-more-FFS(6)				−0.047***	−0.047***	−0.047***
				(0.012)	(0.012)	(0.012)
**Type of illness: illness A (ref)**						
Illness B		−0.039***	−0.039***		−0.013**	−0.013**
		(0.004)	(0.004)		(0.005)	(0.005)
Illness C		−0.013**	−0.013**		0.025***	0.025***
		(0.004)	(0.004)		(0.007)	(0.007)
**Severity of illness: Moderate (ref)**						
Intermediate		0.016*	0.016*		−0.057***	−0.057***
		(0.007)	(0.007)		(0.009)	(0.009)
Severe		0.045***	0.045***		−0.100***	−0.100***
		(0.013)	(0.013)		(0.013)	(0.013)
Age			−0.010			0.003
			(0.006)			(0.006)
Gender (female for ref)			0.029			−0.027
			(0.020)			(0.020)
Education (undergraduats for ref)			0.004			−0.014
			(0.027)			(0.033)
Constant	0.168***	0.165***	0.377**	0.128***	0.176***	0.121
	(0.012)	(0.010)	(0.127)	(0.012)	(0.018)	(0.136)
Observations	8100	8100	8100	5400	5400	5400
Subjects	90	90	90	60	60	60
R^2^	0.096	0.126	0.158	0.017	0.128	0.136

This table shows results from random effects model. The dependent variable is the loss of patient benefit. Panel A (columns 1 to 3) and Panel B (columns 4 to 6) show regression results for DRG-based schemes and FFS-based schemes, respectively. The reference category is DRG in panel A and FFS in panel B. ‘Mix-more-DRG(2)’, ‘Mix-more-DRG(4)’, ‘Mix-more-DRG(6)’, ‘Mix-more-FFS(8)’ and ‘Mix-more-FFS(6)’ are dummy variables for the mixed payment schemes. Additionally, we control for the type of illness and severity of illness with illness ‘A’ and severity of illness ‘moderate’ being the reference categories. The variable’ demographics comprise age, gender and education. Robust standard errors, in parentheses below the coefficients, are clustered at individual subject. *** *p* < 0.001, ** *p* < 0.01, * *p* < 0.05

*DRG* Diagnosis-Related-Group, *FFS* Fee-for-Service

## Discussion

This study examined the effects of FFS, DRG, and mixed payment schemes on physicians’ medical service behavior in a controlled laboratory experiment. By incorporating DRG and several mixed payment schemes, this study adds to the literature on the effects of payment incentives on physicians’ behavior. We simplified DRG as the per capita of multiple disease groups based on the characteristics of DRG payment and designed five different mixed payment schemes; this differed from the experimental designs of some previous studies (e.g., [24, 33]). Our design isolated the effects of payment schemes on physicians’ behavior through keeping all factors potentially affecting physicians’ behavior except for payment schemes constant, which enhanced the robustness of the results.

First, the results supported the underprovision and overprovision of services under pure DRG and FFS payment schemes, respectively, which was consistent with previous studies [32, 33]. Furthermore, mixed payment schemes combining DRG and FFS reduced the underprovision and overprovision observed under pure DRG and FFS schemes, respectively, bringing more benefit to patients than under pure payment schemes; this was consistent with Brosig-Koch et al. [24, 25]. Second, the patient’s health condition affected physicians’ behaviors in different directions under different payment schemes. Namely, a higher disease severity was associated with greater differences between physicians’ quantity choices and q* under DRG and Mix-more-DRG schemes; it was the opposite, however, under FFS and Mix-more-FFS schemes. This suggests that when patients are in lower disease severity and resource consumption is relatively small, prospective payments or mixed systems based on prospective payments are more suitable. When patients have higher disease severity, retrospective payments or mixed systems based predominantly on retrospective payments are better. Third, the data showed that a decrease in payment level did not significantly influence physicians’ behavior and patients’ benefit; this agreed with Brosig-Koch et al. [27] and Keser et al. [39]. This means it is possible to control health expenditures by designing mixed payment schemes that decrease remuneration for physicians. Some studies, however, obtained opposite findings (e.g., [40, 41]) . Thus, there needs to be more researches on how to keep a balance between controlling health expenditures and maintaining medical quality. Fourth, different from Brosig-Koch et al. [24], we found that physicians did not respond to the presentation of payment incentives. Based on the interviews and questionnaires, we learned that although the subjects knew they made decisions under pure and mixed payment schemes in Part 1 and Part 2, respectively, they were more concerned about whether there were substantial changes in physicians’ profit and patients’ benefit with the same or different quantity choices in the two parts of the experiment, rather than the presentation of payment schemes. We also observed altruistic preferences in physicians’ quantity choices under pure and mixed payment schemes. Under the pure and mixed payment schemes, physicians didn’t always choose the quantity that maximized their personal profits, which reflected the altruistic behavior of physicians. And the decrease in physicians’ trade-off between personal profits and patients’ benefit led to less deviations from physicians’ quantity choices to the optimal quantity under mixed payment schemes. This suggests that mixed payment schemes may help motivate less altruistic physicians to choose the optimal quantity for patients [25].

### Limitations

First, by the device of “patient benefit”, we incorporated the real patients in the experiment, as the subjects’ decisions were consequential for real patients to receive medical treatment outside the laboratory (e.g. [23–27, 42]). Compared with pure payment schemes, the higher patients’ benefit under mixed payment schemes indicated that physicians’ decisions were more likely to increase patients’ benefit and improve the quality of care. But given the multidimensionality and complexity of quality of care, mirroring the quality of care by using patient benefit may not fully reflect the impact of payment methods on healthcare quality. And the abstraction of experimental parameters might reduce the external validity of the results. Further, we didn’t introduce a condition that started with mixed payment schemes following by pure payment schemes and our results may be potentially confounded by order effects. The second problem may be related to using medical students in the role of physicians as subjects in our experiment. Brosig-Koch et al. [27, 42] and Reif et al. [43] showed that physicians behaved in a similar way as students did. Brosig-Koch et al. [42] and Reif et al. [43] reported that physicians responded lesser to financial incentives compared to students did, as physicians were more concerned about patients’ benefit. While, Brosig-Koch et al. [27] reported that physicians were more sensitive to the introduction of performance pay than medical students. The results from Wang et al. [44] showed that Chinese physicians seemed to respond stronger to financial incentives than medical students. Considering the differences in these studies and the external validity of the experimental results, we think it still needs to conduct more behavioral experiments involving healthcare professionals to extend our findings from laboratory experiments to field experiments. Third, we investigated the direct effects of payment incentives on physicians’ behavior. While, with the emergence of health maintenance organization and other similar medical organizations, some studies have noted that the internal incentives of medical organizations could influence payment systems’ effects on physicians’ behavior (e.g., [45, 46]). If there is incentive incompatibility between the payment of medical insurance to medical organizations and payment of medical organizations to individual physicians, the incentivizing effects of payment systems cannot be effectively transmitted to physicians’ behavior. Attention should be paid to this aspect in future research on the effects of payment incentives on physicians’ behavior.

## Conclusion

Our results show that the advantages of mixed payment schemes in maintaining good quality care while restraining health expenditures compared to pure payment schemes. That supports the movement from pure payment schemes to mixed payment schemes that blend prospective payments and retrospective payments. Our findings also suggest that the design of mixed payment systems should be adjusted according to the patients’ health conditions. And, unobserved physicians’ behavioral response to the decrease of payment level and physicians’ altruistic preferences indicate that monetary incentives are not always effective. We should pay attention to the influence of non-monetary incentives in the further related studies.

## Supplementary Information

**Additional file 1.** Instructions + Comprehension Questions.

**Additional file 2. **Questionnaire Survey.

**Additional file 3. **Parameter Tables.

**Additional file 4. **Additional analyses of the presentation effects.

## Data Availability

The datasets generated and/or analysed during the current study are not publicly available due to ethical issues but are available from the corresponding author on reasonable request.

## Abbreviations

FFS: Fee-for-service
CAP: Capitation
DRG: Diagnosis-Related-Group
Mix-more-DRG(2), Mix-more-DRG(4), Mix-more-DRG(6): DRG-based mixed payment schemes that has higher DRG weight
Mix-more-FFS(8), Mix-more-FFS(6): FFS-based mixed payment schemes that has higher FFS weight
DRG^pre^: The presentation of pure DRG
FFS^pre^: The presentation of pure FFS
NA-mix-more-DRG(2): Non-adjusted Mix-more-DRG(2)
NA-mix-more-FFS(8): Non-adjusted Mix-more-FFS(8)
WSR test: The matched-pairs Wilcoxon signed-rank test
MWU test: Mann-Whitney U test
KWH test: Kruskal-Wallis H test
SD: Standard Deviation
REM: Random Effects Model
