# Supplementary material for: Effects of fee-for-service, diagnosis-related-group, and mixed payment systems on physicians’ medical service behavior: experimental evidence
Source: BMC Health Serv Res. 2022 Jul 5;22:870. doi: 10.1186/s12913-022-08218-5 (PMC9258053; doi:10.1186/s12913-022-08218-5)

**Additional file 1: Instructions + Comprehension Questions**

Thank you for participating in the experiment on time! You are participating in an economic experiment on decision-making behavior. In the experiment, you, in a role of physician, will provide medical services for different types of patients under different payment schemes. According to your decisions, you will receive a payment after the experiment.

The experiment will last for about 90 minutes and consists of two parts. In Part 1, you will make decisions under pure payment schemes, Diagnosis-related-group (DRG) or fee-for-service (FFS). In Part 2, you will make decisions under mixed payment schemes, which comprises both DRG and FFS in different ratios. The decisions made in either part of the experiment will not affect the other part of the experiment.

Please read the following instructions carefully. If you have any questions, please raise your hand and we will answer any questions you may ask. Please do not talk to others during the experiment. Thank you for your cooperation!

1 Experimental Situations

In the experiment, you will take on the role of a physician and choose the quantity of medical services for different types of patients. The type of every patient is characterized by one of three illnesses (A, B, C), and each illness may occur in three different degrees of severity (moderate (l), intermediate (m), severe (h)). In each decision-making task, you will face one patient type, who is characterized by one of 9 combinations of different illnesses and severities (A_l_, B_l_, C_l_, A_m_, B_m_, C_m_, A_h_, B_h_, C_h_). Your decision is to provide the patient with a quantity of 0, 1, 2, 3, 4, 5, 6, 7, 8, 9 or 10 medical services.

You will receive a remuneration for your medical treatment. In Part 1, you remuneration is irrespective of the quantity of medical services (increases with the quantity of medical services) that you provide in DRG (FFS). In Part 2, you remuneration includes two parts. One part is irrespective of the quantity of medical services (increases with the quantity of medical services) that you provide. The other part increases with the quantity of medical services (is irrespective of the quantity of medical services) that you provide. The calculated total remuneration is given in the experiment. And you will also need to pay for the costs for treating patients, which depends on the quantity of medical services you provide. Your profit of each decision is calculated by subtracting the costs from the payment compensation.

Each quantity of medical services yields a specific health benefit of patient, depending on the patients’ illness and severity. Therefore, your decision will not only affect your profit, but also the patients’ benefit.

2 Payment

All amounts in the experiment are presented in Talers. 1 Taler equal 0.1 CNY. After the experiment, the basic remuneration 30 CNY and your profit in all the experimental decisions will be paid by bank transfer.

Although there are no real patients in the experiment, but your decisions do benefit real patients. One of the five rounds of the experiment will be chosen randomly. The patients’ benefit in this round will be donated to the Red Cross Society of China. Two participants will be selected randomly from all subjects as monitors of donation. And the donation will be carried out by the experimenter and two monitors. Each of the monitor will receive an additional 50 CNY for remuneration.

3 Experimental Steps

Step 1: Experiment Introductions. Please read the experimental instructions. If you have any questions, please raise your hand. Before the formal experiment, we will have a pilot experiment to make you get familiar with this experiment.

Step 2: Experiment Decisions. According to the information presented on the computer screen, you need to choose the quantity of medical services for different types of patients within the specified time. Your operation is to enter a non-negative integer, which values in 0, 1, 2, 3, 4, 5, 6, 7, 8, 9 or 10, on the computer screen.

Step 3: Experiment payments. Your profit and patient benefit of each decision will be displayed on the computer screen.

Step 4: Experiment Rounds. The experiment consists of five rounds and each round including 18 decisions. When all participants complete the current experimental decision, the experiment will enter to the next decision automatically. Repeat the above operations until all experimental decisions are completed.

Pilot experiment Instructions:

The pilot experiment consists of two parts. In Part One, you will need to answer some comprehension questions.

Experimental interface 1 in Part One: The upper left of the experiment interface is the payment scheme and patient type corresponding to each decision, such as “Payment scheme: DRG; Patient type: B_m_”. The middle part of the experiment interface, presents various information, “Your Payment”, “Your Cost”, “Your Profit” and “Patient Benefit”, which are corresponding to different medical services you provide.

The questions you need to answer are as follows:

Assuming that the quantity of medical services you provide is 4, then:

(1) What is the “Your payment”?

(2) What is the “Your Cost”?

(3) What is the “Your Profit”?

(4) What is the “Patient Benefit”?

Please fill in the answers to the above questions in the four boxes at the bottom of the experiment interface according to the information on the computer screen. If your answers are correct, after clicking the “OK” button at the bottom right corner, the experiment interface will automatically enter to the Experimental interface 2 in Part One. Otherwise, please re-answer until the answer is correct.

Experimental interface 2 in Part One: The information on the computer screen is the same as that in Experimental interface 1 in Part One.

The questions you need to answer are as follows:

(1) When the “Your Profit” is the highest, what is the quantity of medical services you need to provide?

(2) When the “Patient Benefit” is the highest, what is the quantity of medical services you need to provide?

Please fill in the answers to the above questions in the two boxes at the bottom of the experiment interface according to the information on the computer screen. If your answers are correct, after clicking the “OK” button at the bottom right corner, the experiment interface will automatically enter to the Part Two. Otherwise, please re-answer until the answer is correct.

Part Two of pilot experiment: the simulation of formal experiment.

In the formal experiment, you first need to fill in your corresponding experiment number. The prompt content on the computer screen is “What is your experiment number” (Everyone corresponds to a specific experiment number. Be careful not to fill in wrong number).

After filling in experiment number, you will enter to the experiment interface. The upper left part of the experimental interface will introduce the payment scheme and patient type of each decision, such as “Payment scheme: DRG; Patient type: B_m_”. The middle part of the experiment interface, presents various information, “Your Payment”, “Your Cost”, “Your Profit” and “Patient Benefit”, which is corresponding to different medical services you provide. According to such information, you need to choose the quantity of medical services. A prompt will appear at the bottom of the experiment interface, “Please indicate the quantity of medical services you wish to provide”. Please enter the quantity of medical services (0, 1, 2, 3, 4, 5, 6, 7, 8, 9 or 10) you want to provide in the box behind the prompt.

After inputting the number, when you click the “OK” button at the bottom right corner of the computer screen, the experiment interface will automatically enter to the experiment result output interface. At this time, two lines of content will appear on the computer screen. The first line is “Your Profit is XX”, and the second line is “Patient Benefit is XX”. In the formal experiment, you need to fill in these data into the corresponding place in the experiment record form. After that, click the “OK” button at the bottom right corner of the computer screen and you will automatically enter to the next decision.

Screen shot for DRG payment


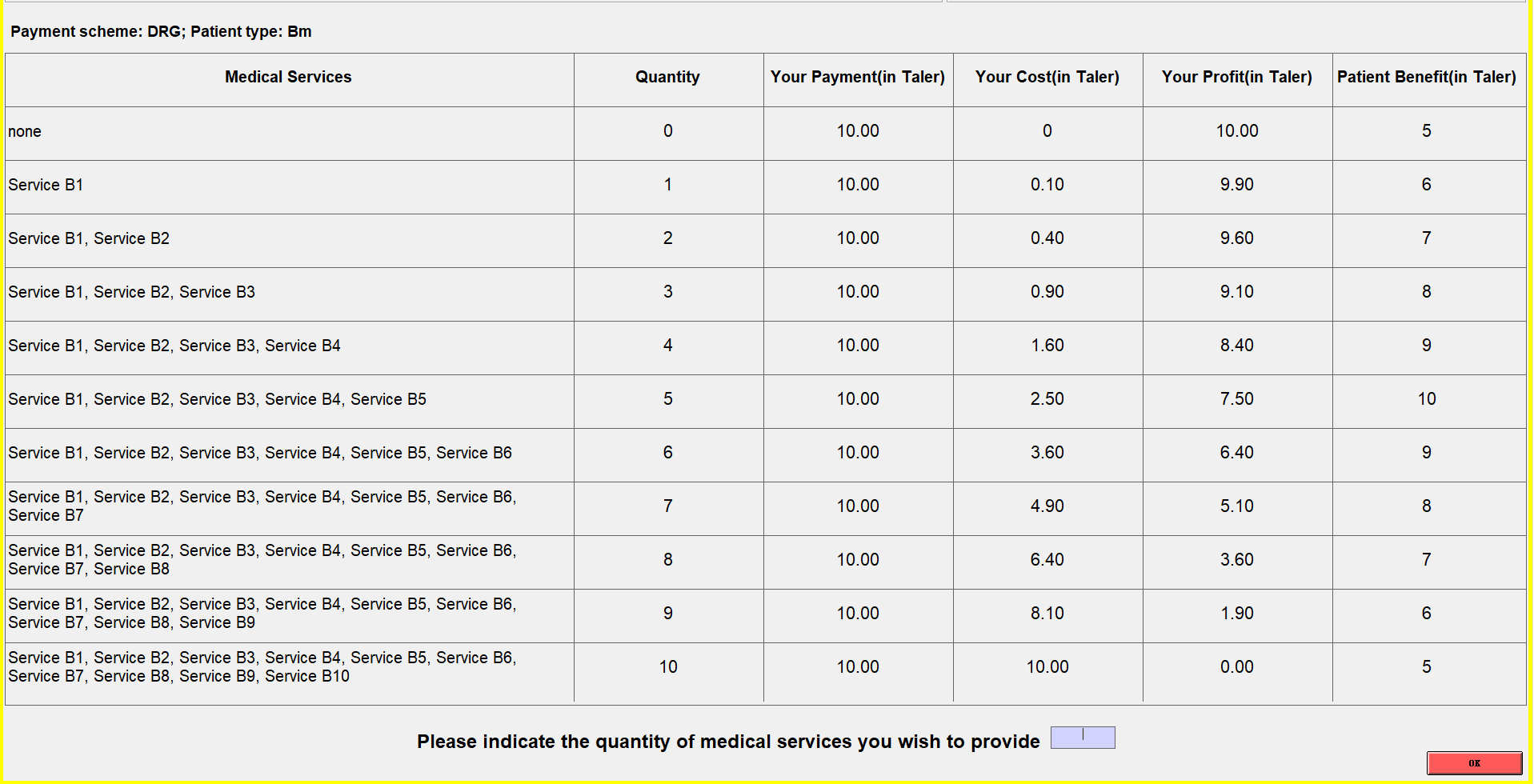

Supplement: Supplementary file 1 — Additional file 1. Instructions + Comprehension Questions. [file 12913_2022_8218_MOESM1_ESM.docx]
