# Supplementary material for: Effects of fee-for-service, diagnosis-related-group, and mixed payment systems on physicians’ medical service behavior: experimental evidence
Source: BMC Health Serv Res. 2022 Jul 5;22:870. doi: 10.1186/s12913-022-08218-5 (PMC9258053; doi:10.1186/s12913-022-08218-5)
Supplement: Supplementary file 2 — Additional file 2. Questionnaire Survey. [file 12913_2022_8218_MOESM2_ESM.docx]

**Additional file 2: Questionnaire Survey**

Payment Methods Economics Experiment Questionnaire

Thank you very much for participating in our economics experiment today. We would like to do a questionnaire survey on the implementation of our economics experiment, and we hope you can participate in this survey. It will take you a few minutes if you would like to participate in this survey. To ensure the validity of the questionnaire, please answer each question truthfully.

(1) How did you know about our economics experiment and sign up?

(2) Why did you come to participate in our experiment?

(3) In the experiment, what aspects did you consider when making decisions? What are the main reasons that make you determine your decisions?

(4) In the hospital, what aspects do you consider when you provide medical services to patients?

(5) Do you have any questions or suggestions regarding the implementation of the experiment?

This survey is over. Thank you again for your cooperation. Wish you have a better academic progress and a happier life!
