# Supplementary material for: Effects of fee-for-service, diagnosis-related-group, and mixed payment systems on physicians’ medical service behavior: experimental evidence
Source: BMC Health Serv Res. 2022 Jul 5;22:870. doi: 10.1186/s12913-022-08218-5 (PMC9258053; doi:10.1186/s12913-022-08218-5)
Supplement: Supplementary file 3 — Additional file 3. Parameter Tables. [file 12913_2022_8218_MOESM3_ESM.docx]

**Additional file 3: Parameter Tables**

Table S1 Experimental Parameters of pure FFS and Mix-more-FFS schemes

| Quantity (q) | | 0 | 1 | 2 | 3 | 4 | 5 | 6 | 7 | 8 | 9 | 10 |
| --- | --- | --- | --- | --- | --- | --- | --- | --- | --- | --- | --- | --- |
| FFS | A_l_ | 0 | 1.81 | 3.42 | 4.83 | 6.04 | 7.05 | 7.86 | 8.47 | 8.88 | 9.09 | 9.10 |
|  | A_m_ | 0 | 1.81 | 3.42 | 4.83 | 6.04 | 7.05 | 7.86 | 8.47 | 8.88 | 9.09 | 9.10 |
|  | A_h_ | 0 | 1.81 | 3.42 | 4.83 | 6.04 | 7.05 | 7.86 | 8.47 | 8.88 | 9.09 | 9.10 |
|  | B_l_ | 0 | 1.90 | 3.60 | 5.10 | 6.40 | 7.50 | 8.40 | 9.10 | 9.60 | 9.90 | 10.00 |
|  | B_m_ | 0 | 1.90 | 3.60 | 5.10 | 6.40 | 7.50 | 8.40 | 9.10 | 9.60 | 9.90 | 10.00 |
|  | B_h_ | 0 | 1.90 | 3.60 | 5.10 | 6.40 | 7.50 | 8.40 | 9.10 | 9.60 | 9.90 | 10.00 |
|  | C_l_ | 0 | 2.00 | 3.80 | 5.40 | 6.80 | 8.00 | 9.00 | 9.80 | 10.40 | 10.80 | 11.00 |
|  | C_m_ | 0 | 2.00 | 3.80 | 5.40 | 6.80 | 8.00 | 9.00 | 9.80 | 10.40 | 10.80 | 11.00 |
|  | C_h_ | 0 | 2.00 | 3.80 | 5.40 | 6.80 | 8.00 | 9.00 | 9.80 | 10.40 | 10.80 | 11.00 |
| Mix-  more-  FFS(8) | A_l_ | 3.43 | 4.84 | 6.05 | 7.06 | 7.86 | 8.47 | 8.88 | 9.09 | 9.10 | 8.91 | 8.52 |
|  | A_m_ | 3.28 | 4.70 | 5.93 | 6.96 | 7.79 | 8.42 | 8.84 | 9.07 | 9.10 | 8.93 | 8.56 |
|  | A_h_ | 3.12 | 4.57 | 5.82 | 6.86 | 7.71 | 8.36 | 8.81 | 9.05 | 9.10 | 8.95 | 8.59 |
|  | B_l_ | 3.76 | 5.24 | 6.52 | 7.60 | 8.48 | 9.16 | 9.64 | 9.92 | 10.00 | 9.88 | 9.56 |
|  | B_m_ | 3.60 | 5.10 | 6.40 | 7.50 | 8.40 | 9.10 | 9.60 | 9.90 | 10.00 | 9.90 | 9.60 |
|  | B_h_ | 3.44 | 4.96 | 6.28 | 7.40 | 8.32 | 9.04 | 9.56 | 9.88 | 10.00 | 9.92 | 9.64 |
|  | C_l_ | 4.13 | 5.69 | 7.05 | 8.21 | 9.16 | 9.92 | 10.48 | 10.84 | 11.00 | 10.96 | 10.72 |
|  | C_m_ | 3.96 | 5.54 | 6.92 | 8.10 | 9.08 | 9.86 | 10.44 | 10.82 | 11.00 | 10.98 | 10.76 |
|  | C_h_ | 3.79 | 5.39 | 6.79 | 8.00 | 9.00 | 9.80 | 10.40 | 10.80 | 11.00 | 11.00 | 10.80 |
| Mix-  more-  FFS(6) | A_l_ | 5.94 | 6.96 | 7.79 | 8.42 | 8.84 | 9.07 | 9.10 | 8.93 | 8.55 | 7.98 | 7.21 |
|  | A_m_ | 5.82 | 6.87 | 7.72 | 8.36 | 8.81 | 9.05 | 9.10 | 8.95 | 8.59 | 8.04 | 7.28 |
|  | A_h_ | 5.71 | 6.77 | 7.64 | 8.30 | 8.77 | 9.04 | 9.10 | 8.97 | 8.63 | 8.10 | 7.36 |
|  | B_l_ | 6.52 | 7.60 | 8.48 | 9.16 | 9.64 | 9.92 | 10.00 | 9.88 | 9.56 | 9.04 | 8.32 |
|  | B_m_ | 6.40 | 7.50 | 8.40 | 9.10 | 9.60 | 9.90 | 10.00 | 9.90 | 9.60 | 9.10 | 8.40 |
|  | B_h_ | 6.28 | 7.40 | 8.32 | 9.04 | 9.56 | 9.88 | 10.00 | 9.92 | 9.64 | 9.16 | 8.48 |
|  | C_l_ | 7.17 | 8.31 | 9.24 | 9.98 | 10.52 | 10.86 | 11.00 | 10.94 | 10.68 | 10.22 | 9.56 |
|  | C_m_ | 7.04 | 8.20 | 9.16 | 9.92 | 10.48 | 10.84 | 11.00 | 10.96 | 10.72 | 10.28 | 9.64 |
|  | C_h_ | 6.91 | 8.10 | 9.08 | 9.86 | 10.44 | 10.82 | 11.00 | 10.98 | 10.76 | 10.34 | 9.72 |

Table S2 Experimental Parameters of pure DRG and Mix-more-DRG schemes

| Quantity (q) | | 0 | 1 | 2 | 3 | 4 | 5 | 6 | 7 | 8 | 9 | 10 |
| --- | --- | --- | --- | --- | --- | --- | --- | --- | --- | --- | --- | --- |
| DRG | A_l_ | 5.73 | 5.63 | 5.33 | 4.83 | 4.13 | 3.23 | 2.13 | 0.83 | -0.67 | -2.37 | -4.27 |
|  | A_m_ | 9.55 | 9.45 | 9.15 | 8.65 | 7.95 | 7.05 | 5.95 | 4.65 | 3.15 | 1.45 | -0.45 |
|  | A_h_ | 13.37 | 13.27 | 12.97 | 12.47 | 11.77 | 10.87 | 9.77 | 8.47 | 6.97 | 5.27 | 3.37 |
|  | B_l_ | 6.00 | 5.90 | 5.60 | 5.10 | 4.40 | 3.50 | 2.40 | 1.10 | -0.40 | -2.10 | -4.00 |
|  | B_m_ | 10.00 | 9.90 | 9.60 | 9.10 | 8.40 | 7.50 | 6.40 | 5.10 | 3.60 | 1.90 | 0.00 |
|  | B_h_ | 14.00 | 13.90 | 13.60 | 13.10 | 12.40 | 11.50 | 10.40 | 9.10 | 7.60 | 5.90 | 4.00 |
|  | C_l_ | 6.30 | 6.20 | 5.90 | 5.40 | 4.70 | 3.80 | 2.70 | 1.40 | -0.10 | -1.80 | -3.70 |
|  | C_m_ | 10.50 | 10.40 | 10.10 | 9.60 | 8.90 | 8.00 | 6.90 | 5.60 | 4.10 | 2.40 | 0.50 |
|  | C_h_ | 14.70 | 14.60 | 14.30 | 13.80 | 13.10 | 12.20 | 11.10 | 9.80 | 8.30 | 6.60 | 4.70 |
| Mix-  more-  DRG(2) | A_l_ | 5.33 | 5.63 | 5.73 | 5.63 | 5.32 | 4.82 | 4.12 | 3.22 | 2.12 | 0.82 | -0.68 |
|  | A_m_ | 9.15 | 9.45 | 9.55 | 9.45 | 9.15 | 8.65 | 7.95 | 7.05 | 5.95 | 4.65 | 3.15 |
|  | A_h_ | 12.97 | 13.27 | 13.37 | 13.27 | 12.97 | 12.47 | 11.77 | 10.87 | 9.77 | 8.47 | 6.97 |
|  | B_l_ | 5.60 | 5.90 | 6.00 | 5.90 | 5.60 | 5.10 | 4.40 | 3.50 | 2.40 | 1.10 | -0.40 |
|  | B_m_ | 9.60 | 9.90 | 10.00 | 9.90 | 9.60 | 9.10 | 8.40 | 7.50 | 6.40 | 5.10 | 3.60 |
|  | B_h_ | 13.60 | 13.90 | 14.00 | 13.90 | 13.60 | 13.10 | 12.40 | 11.50 | 10.40 | 9.10 | 7.60 |
|  | C_l_ | 5.90 | 6.20 | 6.30 | 6.20 | 5.90 | 5.40 | 4.70 | 3.80 | 2.70 | 1.40 | -0.10 |
|  | C_m_ | 10.10 | 10.40 | 10.50 | 10.40 | 10.10 | 9.60 | 8.90 | 8.00 | 6.90 | 5.60 | 4.10 |
|  | C_h_ | 14.30 | 14.60 | 14.70 | 14.60 | 14.30 | 13.80 | 13.10 | 12.20 | 11.10 | 9.80 | 8.30 |
| Mix-  more-  DRG(4) | A_l_ | 4.13 | 4.83 | 5.33 | 5.63 | 5.73 | 5.63 | 5.33 | 4.83 | 4.13 | 3.23 | 2.13 |
|  | A_m_ | 7.95 | 8.65 | 9.15 | 9.45 | 9.55 | 9.45 | 9.15 | 8.65 | 7.95 | 7.05 | 5.95 |
|  | A_h_ | 11.77 | 12.47 | 12.97 | 13.27 | 13.37 | 13.27 | 12.97 | 12.47 | 11.78 | 10.88 | 9.78 |
|  | B_l_ | 4.40 | 5.10 | 5.60 | 5.90 | 6.00 | 5.90 | 5.60 | 5.10 | 4.40 | 3.50 | 2.40 |
|  | B_m_ | 8.40 | 9.10 | 9.60 | 9.90 | 10.00 | 9.90 | 9.60 | 9.10 | 8.40 | 7.50 | 6.40 |
|  | B_h_ | 12.40 | 13.10 | 13.60 | 13.90 | 14.00 | 13.90 | 13.60 | 13.11 | 12.41 | 11.51 | 10.41 |
|  | C_l_ | 4.70 | 5.40 | 5.90 | 6.20 | 6.30 | 6.20 | 5.90 | 5.40 | 4.70 | 3.80 | 2.70 |
|  | C_m_ | 8.90 | 9.60 | 10.10 | 10.40 | 10.50 | 10.40 | 10.10 | 9.60 | 8.90 | 8.00 | 6.90 |
|  | C_h_ | 13.10 | 13.80 | 14.30 | 14.60 | 14.70 | 14.60 | 14.30 | 13.80 | 13.10 | 12.20 | 11.10 |
| Mix-  more-  DRG(6) | A_l_ | 2.13 | 3.23 | 4.13 | 4.83 | 5.33 | 5.63 | 5.73 | 5.63 | 5.33 | 4.83 | 4.13 |
|  | A_m_ | 5.95 | 7.05 | 7.95 | 8.65 | 9.15 | 9.45 | 9.55 | 9.45 | 9.15 | 8.65 | 7.95 |
|  | A_h_ | 9.77 | 10.87 | 11.77 | 12.47 | 12.97 | 13.27 | 13.37 | 13.27 | 12.97 | 12.47 | 11.77 |
|  | B_l_ | 2.40 | 3.50 | 4.40 | 5.10 | 5.60 | 5.90 | 6.00 | 5.90 | 5.60 | 5.10 | 4.40 |
|  | B_m_ | 6.40 | 7.50 | 8.40 | 9.10 | 9.60 | 9.90 | 10.00 | 9.90 | 9.60 | 9.10 | 8.40 |
|  | B_h_ | 10.40 | 11.50 | 12.40 | 13.10 | 13.60 | 13.90 | 14.00 | 13.90 | 13.60 | 13.10 | 12.40 |
|  | C_l_ | 2.70 | 3.80 | 4.70 | 5.40 | 5.90 | 6.20 | 6.30 | 6.20 | 5.90 | 5.40 | 4.70 |
|  | C_m_ | 6.90 | 8.00 | 8.90 | 9.60 | 10.10 | 10.40 | 10.50 | 10.40 | 10.10 | 9.60 | 8.90 |
|  | C_h_ | 11.10 | 12.20 | 13.10 | 13.80 | 14.30 | 14.60 | 14.70 | 14.60 | 14.30 | 13.80 | 13.10 |

Table S3 Experimental Parameters in Conditions P-NA-F8 and P-NA-D2

| Quantity (q) | | 0 | 1 | 2 | 3 | 4 | 5 | 6 | 7 | 8 | 9 | 10 |
| --- | --- | --- | --- | --- | --- | --- | --- | --- | --- | --- | --- | --- |
| P-  NA-F8 | A_l_ | 0.47 | 1.97 | 3.27 | 4.37 | 5.27 | 5.97 | 6.47 | 6.77 | 6.87 | 6.77 | 6.47 |
|  | A_m_ | 1.33 | 2.83 | 4.13 | 5.23 | 6.13 | 6.83 | 7.33 | 7.63 | 7.73 | 7.63 | 7.33 |
|  | A_h_ | 2.12 | 3.62 | 4.92 | 6.02 | 6.92 | 7.62 | 8.12 | 8.42 | 8.52 | 8.42 | 8.12 |
|  | B_l_ | 1.10 | 2.60 | 3.90 | 5.00 | 5.90 | 6.60 | 7.10 | 7.40 | 7.50 | 7.40 | 7.10 |
|  | B_m_ | 2.00 | 3.50 | 4.80 | 5.90 | 6.80 | 7.50 | 8.00 | 8.30 | 8.40 | 8.30 | 8.00 |
|  | B_h_ | 2.82 | 4.32 | 5.62 | 6.72 | 7.62 | 8.32 | 8.82 | 9.12 | 9.22 | 9.12 | 8.82 |
|  | C_l_ | 1.80 | 3.30 | 4.60 | 5.70 | 6.60 | 7.30 | 7.80 | 8.10 | 8.20 | 8.10 | 7.80 |
|  | C_m_ | 2.74 | 4.24 | 5.54 | 6.64 | 7.54 | 8.24 | 8.74 | 9.04 | 9.14 | 9.04 | 8.74 |
|  | C_h_ | 3.60 | 5.10 | 6.40 | 7.50 | 8.40 | 9.10 | 9.60 | 9.90 | 10.00 | 9.90 | 9.60 |
| P-  NA-D2 | A_l_ | 4.87 | 5.17 | 5.27 | 5.17 | 4.87 | 4.37 | 3.67 | 2.77 | 1.67 | 0.37 | -1.13 |
|  | A_m_ | 8.52 | 8.82 | 8.92 | 8.82 | 8.52 | 8.02 | 7.32 | 6.42 | 5.32 | 4.02 | 2.52 |
|  | A_h_ | 12.09 | 12.39 | 12.49 | 12.39 | 12.09 | 11.59 | 10.89 | 9.99 | 8.89 | 7.59 | 6.09 |
|  | B_l_ | 5.14 | 5.44 | 5.54 | 5.44 | 5.14 | 4.64 | 3.94 | 3.04 | 1.94 | 0.64 | -0.86 |
|  | B_m_ | 8.96 | 9.26 | 9.36 | 9.26 | 8.96 | 8.46 | 7.76 | 6.86 | 5.76 | 4.46 | 2.96 |
|  | B_h_ | 12.70 | 13.00 | 13.10 | 13.00 | 12.70 | 12.20 | 11.50 | 10.60 | 9.50 | 8.20 | 6.70 |
|  | C_l_ | 5.44 | 5.74 | 5.84 | 5.74 | 5.44 | 4.94 | 4.24 | 3.34 | 2.24 | 0.94 | -0.56 |
|  | C_m_ | 9.45 | 9.75 | 9.85 | 9.75 | 9.45 | 8.95 | 8.25 | 7.35 | 6.25 | 4.95 | 3.45 |
|  | C_h_ | 13.38 | 13.68 | 13.78 | 13.68 | 13.38 | 12.88 | 12.18 | 11.28 | 10.18 | 8.88 | 7.38 |

Table S4 Experimental Parameters of Patient Benefit

| Quantity (q) | | 0 | 1 | 2 | 3 | 4 | 5 | 6 | 7 | 8 | 9 | 10 |
| --- | --- | --- | --- | --- | --- | --- | --- | --- | --- | --- | --- | --- |
| Patient  Benefit | A_l_ | 4 | 5 | 6 | 7 | 6 | 5 | 4 | 3 | 2 | 1 | 0 |
|  | A_m_ | 2 | 3 | 4 | 5 | 6 | 7 | 6 | 5 | 4 | 3 | 2 |
|  | A_h_ | 0 | 1 | 2 | 3 | 4 | 5 | 6 | 7 | 6 | 5 | 4 |
|  | B_l_ | 7 | 8 | 9 | 10 | 9 | 8 | 7 | 6 | 5 | 4 | 3 |
|  | B_m_ | 5 | 6 | 7 | 8 | 9 | 10 | 9 | 8 | 7 | 6 | 5 |
|  | B_h_ | 3 | 4 | 5 | 6 | 7 | 8 | 9 | 10 | 9 | 8 | 7 |
|  | C_l_ | 8 | 10 | 12 | 14 | 12 | 10 | 8 | 6 | 4 | 2 | 0 |
|  | C_m_ | 4 | 6 | 8 | 10 | 12 | 14 | 12 | 10 | 8 | 6 | 4 |
|  | C_h_ | 0 | 2 | 4 | 6 | 8 | 10 | 12 | 14 | 12 | 10 | 8 |
