# Supplementary material for: Effects of fee-for-service, diagnosis-related-group, and mixed payment systems on physicians’ medical service behavior: experimental evidence
Source: BMC Health Serv Res. 2022 Jul 5;22:870. doi: 10.1186/s12913-022-08218-5 (PMC9258053; doi:10.1186/s12913-022-08218-5)
Supplement: Supplementary file 4 — Additional file 4. Additional analyses of the presentation effects. [file 12913_2022_8218_MOESM4_ESM.docx]

**Additional file 4: Additional analyses of the presentation effects**

In order to explore the influence of presentation on physicians’ behavior, we designed the physicians’ profit function and patient benefit function to be the same in Part 1 and Part 2 in groups Ⅵ and Ⅶ. The only difference between the two parts of experiment was which payment scheme was presented to physicians (either DRG (FFS) or a mixed payment scheme). Taking A_l_ as an example, in group Ⅵ, π(q^) under DRG^pre^ and NA-Mix-more-DRG(2) was 5.27 at q = 2; in group Ⅶ, π(q^) under FFS^pre^ and NA-Mix-more-FFS(8) was 6.87 at q = 8. To achieve the above conditions, we also needed to adjust the cost function C_(q)_. In group Ⅵ, 5.73 − C_3_(q) = 5.27 = −0.1·(q − 2)^2^ + 5.27, and the solution is C_3_(q) = 0.1·q^2^ − 0.4·q + 0.86. In group Ⅶ, 1.91·q − C_4_(q) = 6.87 = −0.1·(q − 8)^2^ + 6.87, and the solution is C_4_(q) = 0.1·q^2^ + 0.31·q − 0.47. The other adjusted cost functions could be calculated with reference to the example A_l_.

Table S5 Descriptive statistics for quantity choices

| Group | Experimental Condition | Pure Payment Scheme | | Mixed Payment Scheme | |
| --- | --- | --- | --- | --- | --- |
|  |  | Mean | SD | Mean | SD |
| Ⅵ | P-NA-D2 | 3.96 | 1.67 | 3.98 | 1.65 |
| Ⅶ | P-NA-F8 | 5.91 | 1.60 | 5.89 | 1.62 |

Notes: P-N-D2: the presentation of pure DRG (DRGpre) and non-adjusted Mix-more-DRG(2) (NA-mix-more-DRG(2)). P-N-F8: the presentation of pure FFS (FFSpre) and non-adjusted Mix-more-FFS(8) (NA-mix-more-FFS(8)). The results showed that the presentation of payment incentives did not lead to a change in physicians’ behavior (group Ⅵ, p=0.094>0.05; group Ⅶ, p=0.720>0.05, the matched-pairs Wilcoxon signed-rank (WSR) test).

Table S6 Descriptive statistics for patient benefit

| Group | Experimental Condition | Pure Payment Scheme | | Mixed Payment Scheme | |
| --- | --- | --- | --- | --- | --- |
|  |  | Mean | SD | Mean | SD |
| Ⅵ | P-NA-D2 | 8.93 | 2.92 | 8.97 | 2.90 |
| Ⅶ | P-NA-F8 | 9.00 | 2.93 | 9.01 | 2.99 |

Notes: P-N-D2: the presentation of pure DRG (DRGpre) and non-adjusted Mix-more-DRG(2) (NA-mix-more-DRG(2)). P-N-F8: the presentation of pure FFS (FFSpre) and non-adjusted Mix-more-FFS(8) (NA-mix-more-FFS(8)). Comparing the patient benefit of the two parts, there was no significant difference (group Ⅵ, p=0.107>0.05; group Ⅶ, p=0.932>0.05; WSR test).
